# Statistics of Natural Binocular Images

**DOI:** 10.1068/ig9

**Published:** 2013-10-01

**Authors:** D W Hunter, P B Hibbard

**Affiliations:** University of St Andrews, UK; University of Essex, UK

## Abstract

Understanding exactly how disparity is processed from binocular views of the world is a long standing problem in vision. It has been hypothesised that an important first step in visual processing is to generate an energy efficient sparse coding of visual inputs. This reduces both noise and redundancy. Mathematical models of such codings have been found to produce components that resemble the responses of simple cells in V1. Similar results with binocular inputs suggest that these methods can be used to build hypothetical models of early binocular visual processing. In our study we have used Independent Components Analysis in the vein of work by Hyvärinen and colleagues to find components that form an efficient coding of binocular visual inputs. The distribution of these components was analysed. We found statistical evidence for a bimodal distribution of interocular differences in phase tuning, further strengthening the summation/difference channels theory of Li and Atick (1994). We have also found components tuned to detect mixtures of phase disparity and position disparity in each view similar to the physiological findings of Anzai et al. (1997). This is consistent with the arguments of Read and Cumming (2007) that both phase disparity and position disparity are necessary for stereopsis.

